# The Impact of the Initial Phase of the COVID-19 Pandemic on Patients With Autoimmune Bullous Diseases

**DOI:** 10.7759/cureus.45545

**Published:** 2023-09-19

**Authors:** Sinem Örnek, Betul Erdem, Müzeyyen Gönül

**Affiliations:** 1 Dermatology, Diskapi Yildirim Beyazit Training and Research Hospital, Ankara, TUR

**Keywords:** linear iga bullous disease, bullous pemphigoid, pemphigus vulgaris, autoimmune bullous diseases, covid-19

## Abstract

Introduction

The follow-up of patients with autoimmune bullous diseases (AIBDs) was temporarily interrupted during the initial phase of the COVID-19 pandemic due to restrictions in healthcare services, given the high contagiousness and rapid spread of SARS-CoV-2. Our objective was to assess the impact of the initial phase of the COVID-19 pandemic on the treatments and disease activity of AIBD patients.

Methods

We conducted a telephone survey of patients with AIBDs who had been regularly followed up in our hospital prior to the onset of the pandemic. A structured questionnaire that we designed was used. This questionnaire comprised questions examining the following issues between March and June of 2020: patients' follow-up, treatment, COVID-19 infection status, and changes in disease activity.

Results

Thirty-nine patients were included in the study. Among those, 26 (66.7%) were immunosuppressed. The frequency of follow-up for 37 patients (94.9%) changed significantly (p<0.001): 28 patients (71.8%) did not visit the hospital, and 26 of them (92.9%) did not communicate at all. The treatment for 10 patients (25.6%) was altered, either by their physician or by themselves. Disease activity reactivated in patients who altered their own treatments. There was only one patient (2.6%) who contracted COVID-19.

Conclusions

Documenting this period revealed that some patients were negatively impacted by the pandemic initially. The most significant contributing factor was the interruption of patient-physician communication.

## Introduction

Coronavirus disease 2019 (COVID-19) is a respiratory disease caused by a recently identified virus, severe acute respiratory syndrome coronavirus 2 (SARS-CoV-2). It can be clinically asymptomatic or induce severe viral pneumonia, which may result in respiratory failure and even death [1]. After the World Health Organization (WHO) declared the COVID-19 outbreak a pandemic on March 11, 2020, the health systems of many countries were affected by the pandemic. Healthcare services were reorganized to reduce the spread of the virus and to ensure the safety of patients and healthcare professionals. In Turkey, following the first confirmed case of COVID-19, healthcare services were restricted to only emergencies and cases affected by the COVID-19 outbreak. This led to an interruption in the follow-up of patients with chronic diseases from March to June 2020. One of the patient groups whose follow-up was interrupted consisted of those with autoimmune bullous diseases (AIBDs), a set of rare autoimmune disorders that manifest clinically as erosions and blisters on the skin and mucous membranes [2]. AIBD is commonly managed with immunosuppressive medications that necessitate close clinical monitoring [3]. In our study, we aim to evaluate how the initial phase of the COVID-19 pandemic and the accompanying restrictions on healthcare services impacted the treatments and disease activities of patients with AIBD.

## Materials and methods

Study design

This was a single-center observational study conducted in August 2020 at Diskapi Yildirim Beyazit Training and Research Hospital. The study received approval from the Ethics Committee of Diskapi Yildirim Beyazit Training and Research Hospital with the code 93/03 on August 10, 2020. Approval from the Ministry of Health was also obtained to conduct the study. The study included patients with AIBD who had been regularly followed up in our hospital prior to the onset of the pandemic. A telephone survey was conducted using a structured questionnaire that we developed. Patients who could not be reached by phone were excluded from the study.

The study questionnaire

To assess the impact of the initial phase of the COVID-19 pandemic on patients with AIBD, we developed a structured questionnaire (Appendix). The questionnaire consisted of 16 questions that examined the follow-up, treatment, COVID-19 infection status, and changes in disease activity of the patients between March and June 2020. The questions were pilot-tested on 10 healthy volunteers to ensure comprehensibility, and the final version of the questionnaire was prepared based on the results of the pilot study. Dr. Betul Erdem conducted the surveys and verbally asked questions to each patient during a telephone visit. All patients provided verbal informed consent to participate in the study. Additionally, we collected data on age, gender, duration of disease, diagnosis and treatment of AIBD, pre-pandemic disease activity, follow-up periods, comorbidities, and concurrent treatments from patient files. We categorized patients in "complete remission off therapy," "complete remission on therapy," and "complete remission during tapering" as "being in remission" [4,5].

Data analysis

Statistical analysis was performed using IBM SPSS Statistics for Windows, Version 21 (Released 2012; IBM Corp., Armonk, New York, United States). All investigated features of the study population were analyzed using descriptive statistics. Categorical variables were described using frequencies and percentages. Quantitative variables were presented as mean ± standard deviation (SD) or median (range), as appropriate. The Kolmogorov-Smirnov test was employed to determine the normality of the distribution of numeric variables. The Mann-Whitney U test was used to compare non-normally distributed numeric variables, and the chi-square test or Fisher's exact test was employed to compare categorical variables between independent groups.

## Results

Before the onset of the pandemic, 57 patients with AIBD were regularly followed up at Diskapi Yildirim Beyazit Training and Research Hospital, and 39 of them were reached by telephone and included in the analysis. The demographic and clinical characteristics of the patients are shown in Table 1.

**Table 1:** Demographic and clinic characteristics of the patients

Characteristics	N=39
Age (y), mean ± SD; min-max	53.28 ± 14.30; 18-81
Sex; female, n (%)	27 (69.2)
Duration of disease (mo), mean ± SD; min-max	49.08 ± 60.72; 6-324
Diagnosis, n (%)	
–Pemphigus vulgaris	25 (64.1)
–Bullous pemphigoid	6 (15.4)
–Dermatitis herpetiformis	3 (7.7)
–Linear IgA bullous disease	2 (5.1)
–Pemphigus vegetans	2 (5.1)
–Pemphigus foliaceus	1 (2.6)

Twenty-six patients (66.7%) were immunosuppressed due to AIBD therapy (Table 2). The most common comorbidities and concomitant medications were found to be essential hypertension (35.9%), type 2 diabetes mellitus (28.2%), and antihypertensives and antidiabetics. For others, see Table 3. There was no immunosuppressive agent among the treatments that the patients received due to their comorbidities.

**Table 2:** Treatments associated with autoimmune bullous dermatoses ^a^Prednisolone ≤ 10 mg/day or equivalent ^b^Prednisolone >10 mg/day or equivalent ^c^Dapson or tetracycline or doxycycline or sulfasalazine or nicotinic acid ^d^Azathioprine at a dose of 2-3 mg/kg daily or mycophenolate mofetil at a dose of 2-3 g daily and mycophenolate sodium at a dose of 1.44 mg daily IVIG: intravenous immunoglobulin

Treatment groups	N=39 n (%)
No treatment	4 (10.3)
Low doses of glucocorticosteroids^a^ + other(s)^c^	2 (5.1)
Other(s)^c^	7 (17.9)
Low doses of glucocorticosteroids^a^ + immunosuppresive agent^d^ + other(s)^c^	11 (28.2)
High doses of glucocorticosteroids^b^	2 (5.1)
High doses of glucocorticosteroids^b^ + immunosuppresive agent^d^	10 (25.6)
High doses of glucocorticosteroids^b^ + other(s)^c^	1 (2.6)
High doses of glucocorticosteroids^b^ + IVIG	1 (2.6)
Immunosuppresive agent(s)^d^	1 (2.6)

**Table 3:** Comorbidities and concomitant medications

N=39 Comorbidities, n (%)	Treatments
Essential hypertension, 14 (35.9)	Nebivolol, metoprolol, acetylsalicylic acid, apixaban, perindopril, diltiazem, benidipine, lercanidipine, amlodipine, nifedipine, candesartan plus hydrochlorothiazide, nitrendipine plus enalapril, valsartan plus hydrochlorothiazide, losartan plus hydrochlorothiazide
Diabetes mellitus, 11 (28.2)	Metformin, insulin, dapagliflozin, gliclazide, vildagliptin
Anxiety disorder, 3 (7.8)	Escitalopram, duloxetine, sertraline
Hypothyroidism, 3 (7.8)	Levothyroxine
Hypercholesterolemia, 3 (7.8)	Atorvastatin
Gastritis, 3 (7.8)	Rabeprazole, esomeprazole, lansoprazole
Asthma, 2 (5.1)	Formoterol inhaler
Bipolar disorder, 2 (5.1)	Valproic acid, risperidone, escitalopram, lithium, quetiapine
Depression, 2 (5.1)	Sertraline, duloxetine
Ulcerative colitis, 1 (2.6)	Mesalazine
Epilepsy, 1 (2.6)	Levetiracetam
Sjogren’s syndrome, 1 (2.6)	Hydroxychloroquine
Rheumatoid arthritis, 1 (2.6)	Hydroxychloroquine
Chronic kidney disease, 1 (2.6)	
Chronic pyelonephritis, 1 (2.6)	
Pyostomatitis vegetans, 1 (2.6)	Sulfasalazine
Chronic Hepatitis B virus infection, 1 (2.6)	Tenofovir

The pre-pandemic follow-up periods were every four weeks for 27 patients (69.2%), every eight weeks for nine patients (23.1%), and every two weeks for three patients (7.7%). After the onset of the pandemic, between March and June 2020, the frequency of follow-up for 37 patients (92.6%) changed significantly (p<0.001): 28 patients (75.7%) did not visit the hospital at all, eight patients (21.6%) came less frequently, and one patient (2.7%) came more frequently. Among the patients who did not visit the hospital after the COVID-19 outbreak, two patients (7.1%) contacted their doctor by phone, while 26 of them (92.9%) did not communicate at all.

In the pre-pandemic period, 33 patients (84.6%) were in remission, and eight of them (24.2%) experienced reactivation after the pandemic (p<0.001). Six patients (23.1%) changed their treatments themselves. Two of them (7.7%) stopped their treatments (2 PV), and four (15.4%) decreased their doses (2 PV, 2 BP), resulting in disease reactivation. These patients were those who did not visit the hospital and did not communicate with their doctor during this period. Another four patients whose treatments were changed or discontinued by their doctor remained in remission. The majority of our patients continued to use their medication at doses adjusted during their last face-to-face visits.

One patient (2.6%) contracted a COVID-19 infection: a 74-year-old female with linear IgA bullous disease. Her disease duration was 38 months. In the pre-pandemic period, she was followed up every eight weeks and was in remission, using dapsone. The patient also had hypertension and type 2 diabetes mellitus and was taking relevant medications. After the pandemic, she did not attend follow-up visits but continued to use dapsone. In August, she was hospitalized for two weeks with symptoms of fever (39°C) and chest pain, and a COVID-19 diagnosis was confirmed by PCR test. A thoracic CT scan revealed bilateral scattered pulmonary infiltration. Favipiravir (twice a day) and enoxaparin 6000 IU (once a day) were administered. During the period she was infected with COVID-19, she continued to use dapsone. Her disease activity was not affected by the COVID-19 infection, and she remained in remission after recovery.

## Discussion

AIBDs are a group of skin disorders that are caused by autoreactive antibodies against various intraepidermal and subepidermal adherence proteins, resulting in dysregulation of the immune system and disruption of skin barrier integrity [2]. Patients with AIBDs often require long-term treatment to achieve therapy-off remission and should be closely monitored during treatment [6]. Unfortunately, the COVID-19 pandemic necessitated changes in our dermatology practices. In Turkey, several measures were imposed by the government between March and June 2020, such as enacting curfews and limiting healthcare services to emergencies only [7]. As a result, we observed that the initial phase of the COVID-19 pandemic and healthcare restrictions disrupted the regular follow-ups and hospital visits of our patients with AIBDs and their communication with their doctors. Some patients even altered their treatment without consulting a healthcare professional.

The pandemic raises concerns regarding the management of such diseases. Patients with AIBDs appear to be at risk for a more severe course of COVID-19, as they are often elderly, have comorbidities, and are on chronic immunosuppressive therapy [8, 9]. These factors have been reported as risk factors for severe outcomes of COVID-19 infection [10]. It is also well-known that inappropriate discontinuation of therapy leads to disease reactivation, and uncontrolled disease activity is associated with AIBD-related morbidity and mortality [8,9,11]. Guidelines recommend continuing treatments for patients with AIBDs during the pandemic but also categorize immunosuppressants as high-risk medications that should be suspended in cases of infection [11-13]. Our observations indicate that patients who inappropriately stopped or decreased their medication doses experienced reactivation. We thus suggest that patients should not discontinue their treatments themselves during such a pandemic.

Furthermore, patients who altered their treatments without consulting a healthcare professional were among those who did not communicate with their doctors during the pandemic. Chen et al. [14] reported that patient-physician communication was disrupted at the beginning of the pandemic, much like in our study, and nearly 45% of their patients with AIBDs discontinued their therapy. A meta-analysis found that communication with the physician positively correlates with patient compliance to treatment [15]. Therefore, in situations where face-to-face communication is challenging, alternative methods of communication must be employed to maintain the patient-physician relationship. In Turkey, the use of teledermatology increased during the pandemic compared to the pre-pandemic period [16], proving to be a useful tool for maintaining patient-physician communication.

Finally, our only patient who contracted COVID-19 was on dapsone for disease control and continued the medication throughout her infection, recovering without experiencing any reactivation. Dapsone is unlikely to elevate the risk for a more severe course of infection [11]; however, our patient did not consult her doctor about continuing the medication during her infection. This case underscores the importance of educating patients on the need to maintain communication with their physicians, particularly when dealing with a COVID-19 infection.

## Conclusions

Our study was a single-center study involving a limited number of patients. And, only the patients who could be reached by telephone participated in the study. Nonetheless, our study revealed the negative impact of the initial phase of the pandemic and healthcare restrictions on the patient-physician communication, the importance of this communication for the patients' compliance with their treatment, and the need to inform the patients on the issue. In addition, the fact that patients who discontinued their medications inappropriately experienced reactivation indicates the necessity of communication with their doctors and monitoring during such a pandemic to avoid reactivation.

## Appendices

**Table 4:** Questionnaire to assess the impact of the initial phase of the COVID-19 pandemic on patients with AIBD PCR: polymerase chain reaction, AIBD: autoimmune bullous disease.

23.07.2020
V1
Initials of the patient's surname and name:
Date:
1. Has the COVID-19 outbreak affected how frequently you visit our hospital's private polyclinic for examinations?
–Yes
–No
2. (If you answered "yes" to the previous query) How has the COVID-19 outbreak affected how frequently you visit our hospital's private polyclinic?
–I never came
–I came less often
–I came more often
3. Have you ever had a COVID-19 infection?
–Yes
–No
4. (If you answered "yes" to the previous query) Which month did you have the infection?
–March
–April
–May
–June
–July
–August
5. (If you answered "yes" to the third query) What symptoms have you experienced as a result of COVID-19 infection?
–Fever
–Cough
–Shortness of breath
–Headache
–Diarrhea
–Vomiting
–Other
6. (If you answered "yes" to the third query) How was the COVID-19 infection diagnosed?
–by PCR
–by thorax CT imaging
–by both PCR and thorax CT imaging
–As a result of my symptoms, a suspicion was raised and treatment was initiated.
7. (If you answered "yes" to the third query) What treatment did you receive for COVID-19?
–I received outpatient care.
–I was admitted to an inpatient ward.
–I was admitted to the critical care unit.
–No treatment was given to me.
8. (If you answered "yes" to the third query) Have you ever been in close contact with someone who has COVID-19 infection?
–Yes
–No
9. (If you answered "yes" to the third query) Has anyone in your home become infected with COVID-19?
–Yes
–No
10. (If you answered "yes" to the third query) Has the COVID-19 infection caused an improvement in your skin disease, or has it gotten worse?
–My skin lesions worsened
–My skin lesions improved
–My skin lesions remained unchanged
11. Since the COVID-19 outbreak began, has the treatment you receive for your skin disease changed (whether you are infected or not)?
–Yes
–No
12. (If you answered "yes" to the previous query) What was the alteration in your treatment?
–My prescription was changed. (The medication that was substituted for another will be recorded.)
–My medications were discontinued, and I received no treatment.
–The dosage of my medication was decreased.
–My frequency of medication administration has decreased.
–My medication dosage was increased.
–My frequency of medication administration has increased.
13. (If you answered "yes" to the 11th query) Who altered the skin disease treatment you received?
–Myself
–My doctor
14. (If you answered "yes" to the 11th query) When did the change in your treatment occur?
–Prior to getting the COVID-19 infection.
–After getting the COVID-19 infection.
–I was not infected with the COVID-19 virus.
15. (If you answered "yes" to the 11th query) Has the treatment change caused an improvement in your skin disease, or has it gotten worse?
–My skin lesions worsened
–My skin lesions improved
–My skin lesions remained unchanged
16. (If you answered "yes" to the 11th query) With the beginning of the normalization process on June 1, has your treatment been resumed?
–Yes
–No
